# Identification of differentially expressed genes involved in transient regeneration of the neonatal C57BL/6J mouse heart by digital gene expression profiling

**DOI:** 10.3892/mmr.2014.2109

**Published:** 2014-04-02

**Authors:** MING LIU, JIN-GAI ZHU, ZHANG-BIN YU, GUI-XIAN SONG, YA-HUI SHEN, YAO-QIU LIU, HAI-LANG LIU, CHUN ZHU, LING-MEI QIAN

**Affiliations:** 1 Department of Cardiology, The First Affiliated Hospital of Nanjing Medical University, Nanjing, Jiangsu 210029P.R. China; 2 Department of Pediatrics, Department of ICU, Nanjing Maternal and Child Health Hospital Affiliated to Nanjing Medical University, Nanjing, Jiangsu 210004, P.R. China; 3 State Key Laboratory of Reproductive Medicine, Department of ICU, Nanjing Maternal and Child Health Hospital Affiliated to Nanjing Medical University, Nanjing, Jiangsu 210004, P.R. China

**Keywords:** neonatal mouse, heart, regeneration, digital gene expression profile

## Abstract

Accumulating evidence has revealed that the mammalian heart possesses a measurable capacity for renewal. Neonatal mice retain a regenerative capacity over a short time-frame (≤6 days), but this capacity is lost by 7 days of age. In the present study, differential gene expression profiling of mouse cardiac tissue was performed to further elucidate the mechanisms underlying this process. The global gene expression patterns of the neonatal C57BL/6J mouse heart were examined at three key time-points (1, 6 and 7 days old) using digital gene expression analysis. In the distribution of total clean tags, high-expression tags (>100 copies) were found to be predominant, whereas low expression tags (<5 copies) occupied the majority of distinct tag distributions. In total, 306 differentially expressed genes (DEGs) were detected in cardiac tissue, with the expression levels of 115 genes upregulated and those of 191 genes downregulated in 7-day-old mice compared with expression levels in 1- and 6-day-old mice, respectively. The expression levels of five DEGs were confirmed using quantitative polymerase chain reaction. Gene ontology analysis revealed a large proportion of DEGs distributed throughout the cell, and these DEGs were associated with binding as well as catalytic, hydrolase, transferase and molecular transducer activities. Furthermore, these genes were involved in cellular, metabolic and developmental processes, as well as biological regulation and signaling pathways. Pathway analysis identified the oxidative phosphorylation pathway to be the process most significantly putatively affected by the differential expression of these genes. These data provide the basis for future analysis of the gene expression patterns that regulate the molecular mechanism of cardiac regeneration.

## Introduction

To date, mature cardiac myocytes have been regarded as terminally differentiated cells that are replaced with fibrous scar tissue following injury and have no regenerative capability (1). However, studies have suggested that urodele amphibians and zebrafish possess the capacity for cardiac regeneration (2,3). Furthermore, accumulating evidence has revealed that the mammalian heart possesses a measurable capacity for renewal (4). Mollova *et al* (5) demonstrated that cardiomyocyte proliferation contributes to developmental heart growth in young humans, which suggests that the heart has the capacity to regenerate myocardium in children and adolescents (5). Neonatal mice also retain a capacity for cardiac regeneration over a short time-frame (aged ≤6 days), but this capacity is lost by 7 days of age (6). However, the specific biological mechanisms underlying the process of cardiac regeneration have yet to be elucidated. Furthermore, the mechanisms by which genes and their proteins regulate this process remain unexplored.

A variety of different genes have been identified to be involved in cardiac regeneration (7,8). Kikuchi *et al* (9) reported that GATA binding protein 4 (GATA4) expression increased to stimulate cardiac regeneration following resection, prior to the expression becoming localized to proliferating cardiomyocytes surrounding and within the injury site. Therefore, GATA4 may be a molecular marker of regeneration (9). Recently, Mahmoud *et al* (10) found that overexpression of Meis1 in cardiomyocytes decreased neonatal myocyte proliferation and inhibited neonatal cardiac regeneration. Thus, Meis1 represents a potential therapeutic target for cardiac regeneration (10). Although these general gene expression patterns are meaningful, the global alterations in the expression of genes involved in mammalian cardiac regeneration during the relevant time-period have not been extensively investigated. Therefore, in this study, three key time-points (1, 6 and 7 days old) were selected for the analysis of global gene expression profiles in C57BL/6 mice using the Solexa/Illumina digital gene expression (DGE) system.

## Materials and methods

### Experimental animals and tissue collection

The study was approved by the Animal Care and Use Committee of Nanjing Medical University (Nanjing, China). C57BL/6J male and female mice were purchased from the Model Animal Research Centre of Nanjing University (Nanjing, China) and raised under pathogen-free conditions in individual cases in a temperature-controlled room (18–24°C) with a 12-h light/dark cycle. C57BL/6J females were mated with males at the age of 6 months. The left ventricular apex was removed from neonates 1, 6 and 7 days after birth (6). Specimens (n=5 per group) were snap-frozen in liquid nitrogen and subsequently stored at −80°C. Total RNA was extracted using TRIzol^®^ reagent (Invitrogen Life Technologies, Carlsbad, CA, USA) in accordance with the manufacturer’s instructions.

### DGE-tag profiling

The main reagents and instruments used for RNA library construction and deep-sequencing were the Illumina Gene Expression Sample Prep kit, the Illumina Sequencing Chip (flow cell), the Illumina Cluster Station and the Illumina HiSeq™ 2000 System (Illumina, Inc., San Diego, CA, USA). Sequence tags were prepared using the Illumina DGE-Tag Profiling kit, in accordance with the manufacturer’s instructions. In brief, mRNA was isolated from 6 μg total RNA using magnetic oligo beads. First- and second-strand cDNA was then synthesized using Oligo (dT) primers. The bead-bound cDNA was subsequently digested with *Nla*III, which recognizes and cleaves CATG sites. The fragments [with the exception of the 3′ cDNA fragments connected to the Oligo (dT) beads] were washed away and the Illumina adaptor 1 was ligated to the sticky 5′-end of the digested bead-bound cDNA fragments. The junction of Illumina adaptor 1 and the CATG site is the recognition site of *Mme*I, which has separated recognition and digestion sites. This enzyme then cuts 17 bp downstream of the CATG site, producing tags with adaptor 1. Following the removal of the 3′ fragments using magnetic bead precipitation, Illumina adaptor 2 was ligated to the 3′-ends of the tags, thus producing tags with different adaptors at either end to form a tag library. Following 15 cycles of linear polymerase chain reaction (PCR) amplification, 105-bp fragments were purified by 6% Tris-Borate-EDTA PAGE. Following denaturation, the single-chain molecules were fixed onto the Illumina Sequencing Chip (flow cell). Each molecule then grew into a single-molecule cluster sequencing template through *in situ* amplification. The four types of nucleotides, which were labeled using four different colors, were subsequently added and sequencing was performed using the sequencing by synthesis method. Sequencing-received raw image data were transformed by base calling into sequence data. Raw sequences had 3′ adaptor fragments as well as a few low-quality sequences and several types of impurities. Raw sequences were transformed into clean tags following data processing. All clean tags were aligned to the reference sequences and unambiguous tags were annotated. The number of clean tags corresponding to each gene was counted.

### Determination of gene expression levels and detection of differentially expressed genes (DEGs)

To compare the differential expression of genes across samples, the number of raw clean tags in each library was normalized to the transcripts per million clean tags to obtain normalized gene expression levels. Differential expression of genes across samples was performed using a rigorous algorithm method. Genes were deemed to be significantly differentially expressed with a P-value <0.005, a false discovery rate (FDR) <0.01 and a two-fold relative change threshold in the sequence counts across libraries.

### Gene ontology (GO) and pathway functional enrichment analysis

In gene expression profiling analysis, GO enrichment analysis of functional significance involves the application of a hypergeometric test to map all DEGs to terms in the GO database. This allows the identification of significantly enriched GO terms in DEGs compared with the genome background. Biological functions require the cooperation of different genes. Pathway-based analysis provides information regarding the biological functions of genes and is usually conducted using the Kyoto Encyclopedia of Genes and Genomes, which is the major public pathway-related database. Pathway enrichment analysis identifies significantly enriched metabolic pathways or signal transduction pathways in DEGs compared with the whole genome background. Pathways with Q-values ≤0.05 are considered significantly enriched in DEGs.

### Quantitative PCR (qPCR) analysis

qPCR analysis was used to validate the DGE results. Total RNA was extracted from neonatal mouse heart tissue using TRIzol reagent (Invitrogen Life Technologies). First-strand cDNA was generated with random primers using an AMV Reverse Transcriptase kit (Invitrogen Life Technologies). Specific primers used for PCR were as follows: Dopachrome tautomerase (Dct), forward 5′-GTCCTCCACTCTTTTACAGACG-3′, reverse 5′-ATTCGGTTGTGACCAATGGGT-3′; Gck (glucokinase), forward 5′-ACTTCCCAGGGTCCCACTGCTG-3′, reverse 5′-GGCTTTGGAGGTGCCACGATAG-3′; δ-aminolevulinate synthase 2 (Alas2), forward 5′-ACTCACCGTCTTGGTTCGTCCTC-3′, reverse 5′-TGAGAACAGGTTGGTCCTTGAGTGG-3′; matrix metallopeptidase 12 (Mmp12), forward 5′-CACAACAGTGGGAGAGAAAA-3′, reverse 5′-AGCTTGAATACCAGATGGGATG-3′; perilipin 2 (Plin2), forward 5′-CCCGCAACCTGACCCAGCAG-3′, reverse 5′-CGCCTGCCATCACCCCCAAG-3′. qPCR was performed in an ABI 7300 Sequence Detection system (Applied Biosystems, Foster City, CA, USA) in accordance with the manufacturer’s instructions under the following conditions: Denaturation at 95°C for 10 min, and 40 cycles of 95°C for 15 sec and 60°C for 1 min. GAPDH was used as a normalizer (forward 5′-TTCACCACCATGGAGAAGGC-3′, reverse 5′-GGCATGGACTGTGGTCATGA-3′) and the relative expression levels of genes were calculated according to the 2^−ΔΔCT^ method. Each sample was assayed at least in triplicate and was reproduced at least three times.

### Statistical analysis

Differential mRNA expression was based on the log_2_ ratios between the competing conditions. All data are expressed as the mean ± standard deviation. Statistical analysis was performed using one-way analysis of variance using the SPSS 10.0 statistical software package (SPSS, Inc., Chicago, IL, USA). The threshold of significance was defined as P<0.05.

## Results

### Sequencing quality evaluation

**Figure 1:**
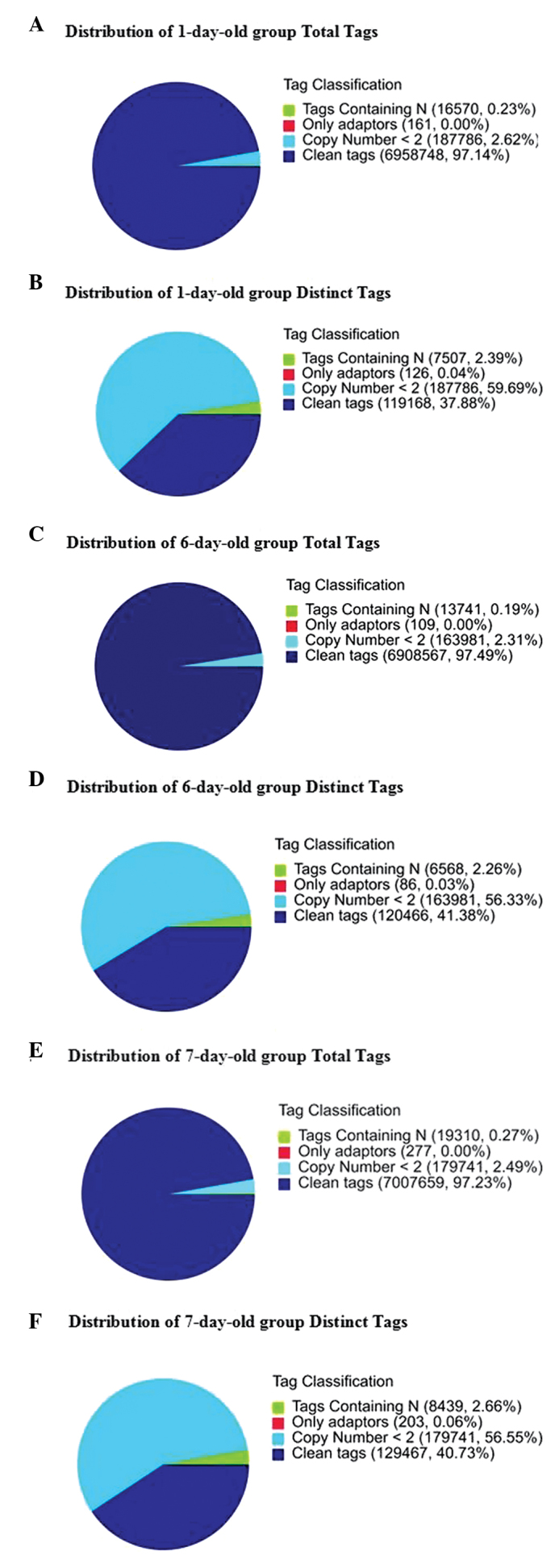
Distribution of tag (total and distinct) expression identified in digital gene expression-tag profiling of mouse cardiac tissue.

**Figure 2:**
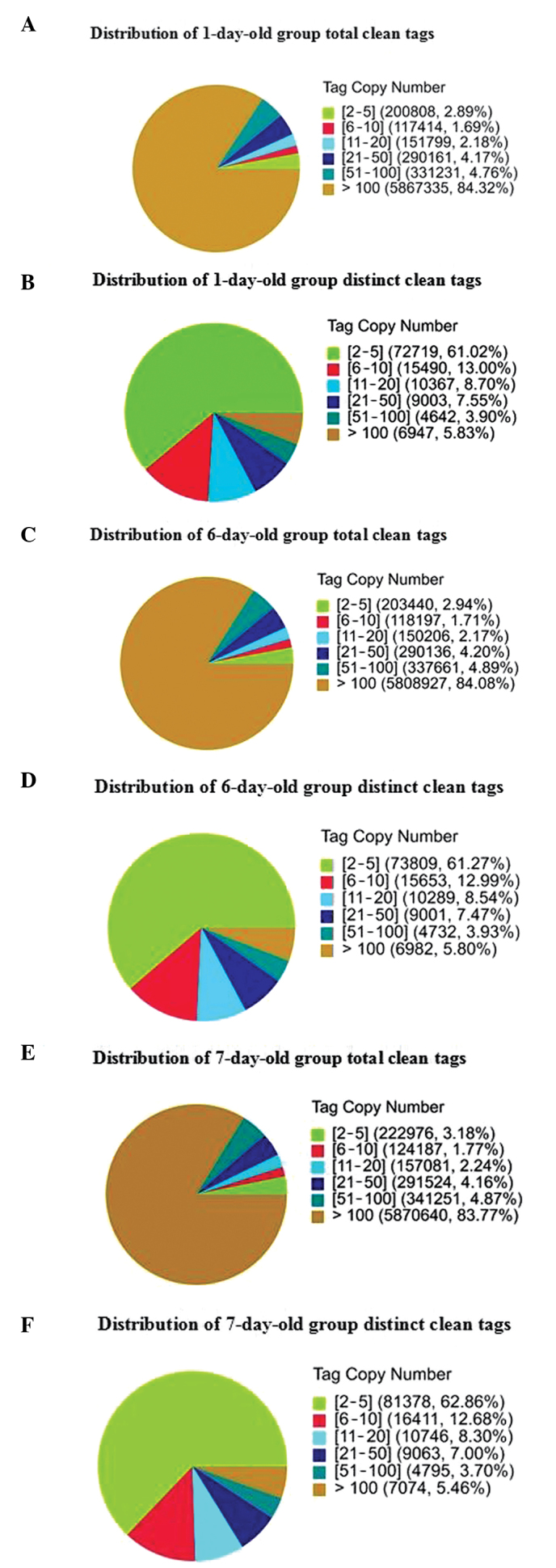
Distribution of clean tag copy number identified in digital gene expression-tag profiling of mouse cardiac tissue.

The number of tags containing clean tags represented 97.14% (6,958,748), 97.49% (6,908,567) and 97.23% (7,007,659) of the total tags in the 1-, 6- and 7-day-old groups, respectively. The number of the distinct tags containing clean tags represented 37.88% (119,168), 41.38% (120,466) and 40.73% (129,467) of the total distinct tags in the three groups, respectively (Fig. 1). The distribution of the total and distinct clean tag copy numbers is shown in Fig. 2. Among the distribution of total clean tags, >80% had copy numbers >100 and <4% had copy numbers between two and five in the three groups. However, the low expression tags (<5 copies) had the highest ratio of the number of distinct to total tags and the lowest percentage of distinct clean high copy number tags. Heterogeneity and redundancy are two significant characteristics of mRNA expression. Certain categories of mRNA have very high abundance, while the majority remain at very low levels of expression. The distribution of clean tag expression may be used to evaluate the normality of the whole data.

### Comparison of gene expression levels among the three groups

The rigorous algorithm method was applied to identify DEGs from the normalized DGE data using pairwise comparisons across all time-points. FDRs were ≤0.01 and estimated absolute |log_2_Ratio| values were ≥1 in at least one of the pairwise comparisons. In detail, 635 upregulated and 738 downregulated genes were identified in 6-day-old mice cardiac tissue samples when the samples were compared with those from 1-day-old mice. Compared with samples from 1-day-old mice, 783 genes were upregulated and 775 genes were downregulated in 7-day-old mice cardiac samples. A total of 902 DEGs were identified in the 7- and 6-day-old mice cardiac tissue samples, including 521 upregulated and 381 downregulated genes. In addition, a total of 306 DEGs, including 115 upregulated and 191 downregulated genes, were detected in 7-day-old cardiac tissue samples compared with samples from 1- and 6-day-old mice, respectively (Table I).

### Validation of DEGs using qPCR

**Figure 3:**
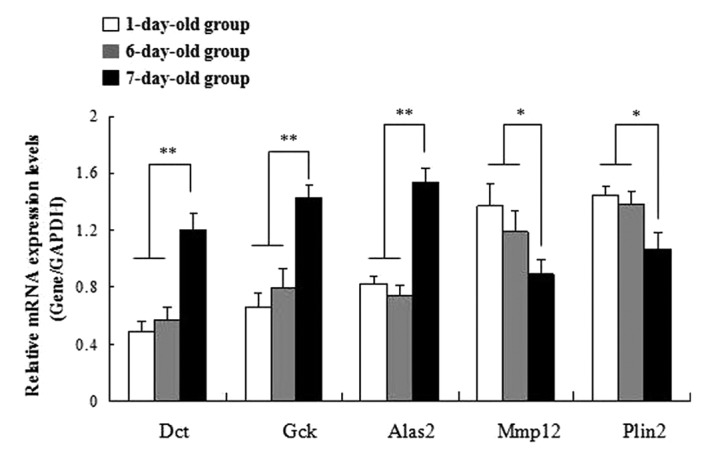
Validation of differentially expressed genes in mouse cardiac tissue using quantitative polymerase reaction. Error bars indicate standard error. ^*^P<0.05; ^**^P<0.01. Dct, dopachrome tautomerase; Gck, glucokinase; Alas2, δ-aminolevulinate synthase 2; Mmp12, matrix metallopeptidase 12; Plin2, perilipin 2.

In order to confirm the DGE results, five genes were selected for qPCR analysis. Among these genes, three (Dct, Gck and Alas2) were upregulated and two (Mmp12 and Plin2) were downregulated in 7-day-old cardiac tissue compared with tissue from 1- and 6-day-old mice. The results of the qPCR were consistent with the DGE analysis (Fig. 3).

### GO and pathway enrichment analysis of DEGs

**Figure 4:**
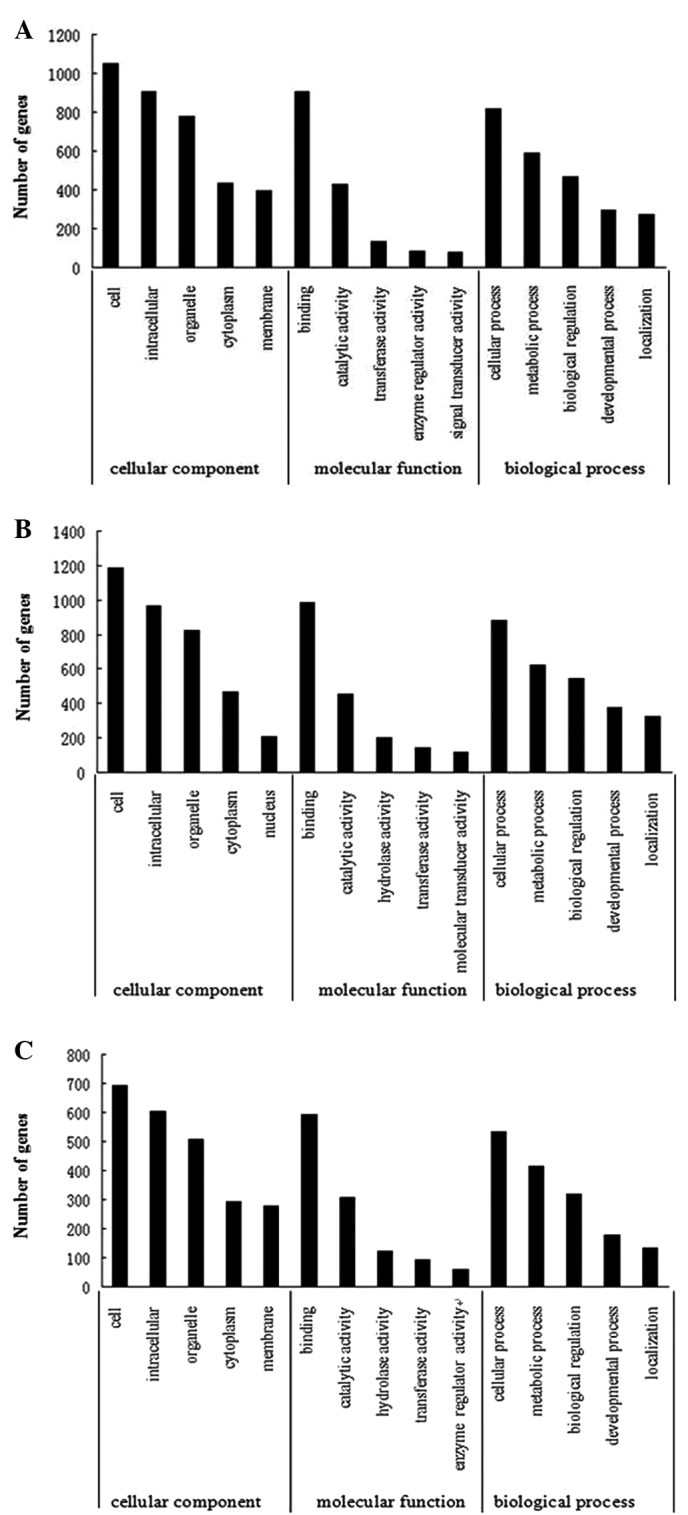
Assignment of differentially expressed genes in mouse cardiac tissue to gene ontology functional categories: (A) 1 vs. 6 days old, (B) 1 vs. 7 days old; (C) 6 vs. 7 days old.

GO has three structured vocabularies: Cellular components, molecular function and biological processes. The basic unit of GO is the GO term. The top five GO terms in this study are shown in Fig. 4. Pathway enrichment analysis revealed the oxidative phosphorylation pathway to be the process that was most significantly putatively affected by the differential expression of these genes in 7-day-old cardiac tissue compared with tissue from 1- and 6-day-old mice (Table II).

## Discussion

In the present study, the global gene expression profiles in regenerative hearts from neonatal mice were analyzed using the Solexa/Illumina DGE system, a tag-based novel high-throughput transcriptome deep-sequencing method. Significant differences in gene expression profiles were observed in cardiac tissues among the three time-points analyzed (1, 6 and 7 days). In this study, tag-mapped genes were confirmed using qPCR. Although the differences in gene expression did not match the magnitude of those detected by the Solexa-based sequencing method, the trends of up- and downregulation were similar. Functional roles for DEGs identified in this study were categorized according to cellular process, regulation of biological processes and metabolic process, thus indicating that multiple genes are involved in the complex biological alterations that occur during cardiac regeneration.

Pathway-Express analysis identified that the oxidative phosphorylation pathway was most significantly affected by the differential expression of genes in 7-day-old cardiac tissue compared with tissue from 1- and 6-day-old mice. Oxidative phosphorylation is a metabolic pathway that uses energy released by the oxidation of nutrients to produce adenosine triphosphate (ATP). Myocardial energy is generated mainly through mitochondrial oxidative phosphorylation (11,12). Various studies have investigated the role of oxidative phosphorylation in liver regeneration, and it has been proposed that oxidative phosphorylation is enhanced following partial hepatectomy in order to compensate for the loss of tissue (13,14). Neonatal mice retain the capacity for cardiac regeneration, but this ability is lost after 7 days. The active biosynthesis of components such as DNA and protein requires energy; therefore, the demand for ATP production in the regenerating heart is increased during the 6 days after birth. It is apparent that the oxidative phosphorylation pathway also has an important role in cardiac development, although the collaborative network of mitochondria-related functions and genes requires further investigation.

Further analysis of the DEGs revealed that MAP kinase phosphatase, Tau, Deltex and Notch were upregulated, and p21 protein (Cdc42/Rac)-activated kinase 1/2, filamin A, α, Dv1 and Delta were downregulated in 7-day-old cardiac tissue compared with tissue from 1- and 6-day-old mice. These are key genes in the mitogen-activated protein kinase (MAPK) and Notch signaling pathways. Previous studies have shown that signaling pathways, including MAPK and Notch signaling pathways, have non-redundant roles in the regulation of zebrafish cardiac regeneration (15–17). It was proposed in the present study that these two signaling pathways participate in mammalian cardiac regeneration, although the collaborative network requires further investigation.

In conclusion, the results of the present study identified changes in the expression of a large number of genes associated with a variety of molecular functions during the first seven days after birth in the neonatal mouse heart. Numerous genes of known and unknown function are involved in mammalian cardiac regeneration, and biochemical and physiological investigations of these genes are likely to be performed in the future.

## Figures and Tables

**Table I:** Differentially expressed genes in neonatal mouse cardiac tissue.

	Age		
	
Genes	1 vs. 6 days (n)	1 vs. 7 days (n)	6 vs. 7 days (n)
Total	1,373	1,558	902
Upregulated	635	783	521
Downregulated	738	775	381

**Table II:** Results from the pathway enrichment analysis.

Age	Pathway	DEGs with pathway annotation, n=1,041 (n)	P-value	Q value
1 vs. 6 days	Cell cycle	23	0.0002558345	0.02855462
	Proteasome	11	0.0002693832	0.02855462
1 vs. 7 days	Oxidative phosphorylation	25	1.098219×10^−5^	0.001646858
	Parkinson’s disease	28	1.546346×10^−5^	0.001646858
6 vs. 7 days	Alzheimer’s disease	26	8.94506×10^−6^	0.001789012
	Parkinson’s disease	18	0.0001735654	0.013760740
	Metabolic pathways	100	0.0002064111	0.013760740
	Oxidative phosphorylation	15	0.0005363196	0.026815980
	Huntington’s disease	23	0.0009825545	0.039302180

DEG, differentially expressed gene.
